# Building a multistate model from electronic health records data for modeling long-term diabetes complications

**DOI:** 10.1017/cts.2024.583

**Published:** 2024-09-23

**Authors:** Riza C. Li, Shanshan Ding, Kevin Ndura, Vishal Patel, Claudine Jurkovitz

**Affiliations:** 1 iREACH, ChristianaCare Health Services, Inc., Newark, DE, USA; 2 Center for Bioinformatics and Computational Biology, University of Delaware, Newark, DE, USA; 3 Department of Applied Economics and Statistics, University of Delaware, Newark, DE, USA

**Keywords:** Diabetes, electronic health records, multistate modeling, diabetes complications, transition probability

## Abstract

**Objective:**

The progression of long-term diabetes complications has led to a decreased quality of life. Our objective was to evaluate the adverse outcomes associated with diabetes based on a patient’s clinical profile by utilizing a multistate modeling approach.

**Methods:**

This was a retrospective study of diabetes patients seen in primary care practices from 2013 to 2017. We implemented a five-state model to examine the progression of patients transitioning from one complication to having multiple complications. Our model incorporated high dimensional covariates from multisource data to investigate the possible effects of different types of factors that are associated with the progression of diabetes.

**Results:**

The cohort consisted of 10,596 patients diagnosed with diabetes and no previous complications associated with the disease. Most of the patients in our study were female, White, and had type 2 diabetes. During our study period, 5928 did not develop complications, 3323 developed microvascular complications, 1313 developed macrovascular complications, and 1129 developed both micro- and macrovascular complications. From our model, we determined that patients had a 0.1334 [0.1284, .1386] rate of developing a microvascular complication compared to 0.0508 [0.0479, .0540] rate of developing a macrovascular complication. The area deprivation index score we incorporated as a proxy for socioeconomic information indicated that patients who reside in more disadvantaged areas have a higher rate of developing a complication compared to those who reside in least disadvantaged areas.

**Conclusions:**

Our work demonstrates how a multistate modeling framework is a comprehensive approach to analyzing the progression of long-term complications associated with diabetes.

## Highlights

- **What is already known on this topic:** The burden that diabetes mellitus presents because of long-term complications not only affect the patients’ health but also their life expectancy.
- **What this study adds:** This study implements a multistate modeling approach to predict micro- or macrovascular complications occurrence and death for an individual based on their specific clinical characteristics at different time periods after diabetes diagnosis.
- **How this study might affect research, practice, or policy:** A multistate modeling approach to diabetes complications can help understand the progression of complications specific to each patient, which will not only aid a physician’s ability to better tailor care but also anticipate complications and plan interventions to reduce the patient’s risk of an event.

## Introduction

In 2020, the Centers for Disease Control and Prevention estimated that 34.1 million adults aged 18 years or older had diabetes mellitus (DM) in the United States (US) population [1]. Complications associated with DM can be categorized into two broad categories, microvascular and macrovascular [2]. Microvascular complications include nephropathy, neuropathy, and retinopathy. Macrovascular complications include cardiovascular disease, stroke, and peripheral vascular disease. Microvascular and macrovascular complications lead to increased mortality and an overall decreased quality of life in individuals with DM. With the burden of DM and its complications, researchers have been investigating predictive analytic methods such as machine learning to evaluate adverse outcomes likelihood according to patients’ clinical profiles.

The widespread adoption of electronic health record (EHR) systems in clinical settings has increased the secondary use of EHR data for predictive analytic models. EHRs are a reliable source of longitudinal observations for monitoring the progression of diseases in clinical practice. EHRs provide large quantities of information regarding a patient’s medical history, including symptoms, examination findings, test results, prescriptions, and procedures. Predictive models play an increasingly important role in the practice of medicine as clinical care becomes more tailored to individual characteristics and needs and precision medicine becomes the norm [3].

Numerous models have already been developed to predict DM complications. Most of the modeling techniques include logistic regression, Cox Proportional Hazard regression, and machine learning techniques such as neural networks and simulation models [4–7]. While the standard Cox model and other machine learning methods have been applied to predict DM complications, these models have been limited in predicting only one event of interest at a time and failed to give an understanding of what happens after the event of interest occurs. Multistate models extend standard time-to-event analysis, offering a more comprehensive process that can describe the progression of a patient through various states [8,9]. The main advantage of implementing a multistate model over traditional time-to-event models is the ability to consider multiple events at the same time and analyze the process of progressing from one event to another. The transition probabilities derived from a multistate model provide the probability of a patient being in a certain state at a specific point in time. For example, Jia et al applied a multistate approach to examine the transition of symptom severity in a cohort of cancer patients in Ontario and demonstrated that symptoms deteriorated over time due to a combination of factors [10].

There have been several studies that have used multistate models to investigate the development of DM complications [11–13]. In one study, researchers collected data over a 25-year time period and applied a multistate model to investigate the transition of type 2 diabetes patients through several complications including retinopathy, coronary artery disease, and microalbuminuria [11]. Another study reported that hemoglobin A1c (HbA1c), systolic blood pressure, and duration of diabetes contributed to the development of microvascular complications in patients with type 1 diabetes [12]. One other study focused on the progression of DM foot disease and its associated risk factors [13]. Although multistate modeling has been employed to predict the progression of DM complications in several studies, only a limited number of risk factors were included in their models. Other limitations included focusing on either type 1 or type 2 diabetes patients exclusively [12]. Finally, these studies restricted their multistate model to only include one complication, such as microvascular complications, or limited their model to a few of the micro- and macrovascular complications [11,13].

The aim of our study was to use a multistate modeling approach to determine the probability of micro- or macrovascular complications occurrence and death in a population of both type 1 and type 2 diabetes patients. We categorized each complication associated with DM as either microvascular or macrovascular to incorporate all complications in our model. We also combined data from multiple sources to explore more features that contribute to the progression of DM complications. Our features are a combination of demographic, clinical, and socioeconomic information taken from EHRs and the Area Deprivation Index (ADI) [14].

## Research design & methods

This study was approved by the ChristianaCare Health Services Inc. Institutional Review Board CCC #38117. ChristianaCare Health Services Inc. is one of the largest health care providers in the mid-Atlantic region, serving most of Delaware and parts of Pennsylvania, Maryland, and New Jersey.

### Study design

This was a retrospective longitudinal study using EHR of patients from ChristianaCare primary care practices and endocrinologist specialists during the period of January 1, 2013, through December 31, 2017, and followed through December 31, 2019. We defined an *Index Visit* as the date of the first ambulatory visit during our study period.

### Study population

Patients who had been diagnosed with the International Classification of Diseases *Ninth Revision* and *Tenth Revision* (ICD9/10) codes for DM were included in the study. We excluded patients less than 18 years of age. Because our goal was to predict the onset of new complications, we excluded patients diagnosed with ICD9/10 DM complications, ICD9/10 DM-related complications, and DM complications-related current procedural terminology (CPT) codes prior to our study period. Patients with no follow-up ambulatory visits after the index visit, and no HbA1c at Index Visit and any follow-up visits were excluded. Patients with both a micro- and macrovascular complication coded on the same visit were excluded because we could not determine which complication occurred first. Lastly, patients who had their first DM diagnosis at *Index Visit* and were not prior ChristianaCare patients were also excluded. We confirmed prior patients by checking if they had prior visits in both ambulatory and hospital at least six months prior to the *Index Visit*. If prior visits were found, we concluded that they were ChristianaCare patients, and the *Index Visit* was the first diagnosis of DM.

### Variables

Our predictor variables include five different categories (Supplement Table 1) from multisource datasets. Demographics include age, sex, race, ethnicity, insurance, diabetes type, smoking status, and duration of diabetes. Vitals include body mass index (BMI), blood pressure systolic, blood pressure diastolic, and heart rate. We had a total of 21 clinical lab variables and 25 Elixhauser comorbidity measures [15]. We also added the ADI as a proxy for socioeconomic status [16]. ADI ranks census block groups for each state from 1 (least disadvantaged neighborhood) to 10 (most disadvantaged neighborhood) [14]. In order to assign a census block group number to a patient, we used the most current address in the EHR and geocoded their location to a specific census block group. The 12-digit census block group code was linked to the latest ADI version. We clustered the decile rankings into five categories of two for simplification.

### Outcomes

Our events of interest are the two types of complications (micro- and macrovascular) associated with DM and death. Complications were defined using ICD9/10 diagnosis codes associated with DM specific complications, ICD9/10 diagnosis codes related to DM complications and CPT codes related to DM complications [17]. The ICD9/10 and CPT codes used to define the complications are provided in Supplement Table 2. Because coding for nephropathy is not very sensitive, we also included the biological definition of chronic kidney disease (CKD) to identify nephropathy [18]. CKD was ascertained if patients had 2 glomerular filtration rate estimates < 60 mL/min/m^2^ at intervals of 90 days or more or 2 abnormal albuminuria/proteinuria at intervals of 90 days or more [19].

### Data preprocessing and challenges

Although EHRs are rich in data, most of these data are not collected in a systematic manner and are organized in multi-dimensional tables resulting in a large number of missing values. In our baseline data (Index visit), we had missing values for smoking status (0.2%), vitals (1.0-10.1%), clinical labs (14.8%-52.8%), and ADI (7.4%). To avoid excluding patients with missing values, we used two different multiple imputation techniques. First we applied the multiple imputation using changed equations (MICE) to our baseline data. MICE has the ability to capture the uncertainty around the imputed values by replacing each missing observation with a set of plausible values yielding multiple imputed datasets. We assumed that the data were missing at random. We used a linear regression model for continuous variables, a logistic regression model for binary variables and a polytomous logistic regression model for categorical variables. The algorithm works by iteratively imputing the missing values based on the fitted conditional models until a stopping criterion is satisfied. With these techniques, missing data for a subject is imputed by a value that is predicted using the subject’s other, known characteristics. We imputed 10 data sets and selected the set that deviated the least from the mean of original data variables [20,21].

Second we used the last observation carry forward (LOCF) technique for our follow-up data, a method applicable to longitudinal or repeated time-series data [22]. Using LOCF, a missing observation is replaced by the most recent observed value for a variable for each patient.

### Statistical analysis

A Markov multistate model was used to examine the process of a disease [9,23,24]. The model contains several transitions and states. States represent the status of a patient at a given time and transitions between states represent a change in a patient status. Patients can transition from a finite number of states during any given time during the observation period. Under the Markov assumption, given an observed state, the future state depends on the present state, but not on the earlier states [25]. In a multistate model, transition intensities represent the instantaneous risk of moving from one state to another. The transition intensity is computed jointly for all possible transitions using a maximum likelihood estimation approach and results in a transition intensity matrix. To understand the effect of covariates on transitions, covariates were fitted using a proportional hazards model to the transition intensity matrix [26]. Additionally multistate models can allow different sets of covariates to be used in modeling different transitions to increase flexibility and reduce the number of parameters in the model. In our model, we employed a variable selection technique to select which variables were included at each transition. A hazard ratio (HR) for each covariate was calculated to analyze the covariate’s effect on each transition. From the transition intensity matrix, it is possible to compute the transition probability matrix within a given period. The transition probability matrix was calculated for one, three, and five years.

A multistate model was built to examine the development of long-term complications after DM diagnosis. We assumed intermittent observation, where the exact date of the transition is unknown but occurred between two observation periods. The only exact known date is the death date. We used the Social Security Death Index database to obtain the exact death dates. We categorized a complication as either a micro- or macrovascular to minimize the complexity of modeling each complication individually. During our study period, a patient occupied one of five distinct states: DM State if a patient did not develop any complications; Microvascular State if a patient developed nephropathy, neuropathy, or retinopathy; Macrovascular State if a patient developed cardiovascular, foot (non-ulcer) and other complications; Both State if a patient developed a macrovascular complication following a previous microvascular complication diagnosis and vice versa; Death State if a patient died during the follow-up period. Patients could only progressively move forward through the states and could transition to the Death State from any of the other states. We did not allow backward transitions (Micro/Macro State to DM State) since a complete reversal of long-term complications is not always possible even with proper management and care. We did not allow the transition from DM State directly to Both State. Since we were following the progression of a patient moving from one DM complication to the other, one at a time, to calculate the transition rate, patients were excluded if we could not determine which type of complication occurred first. In a multistate model, censoring is often considered at the state level, but not at the time of the event. In this case, censoring means that we do not know (observe) the exact state of a patient (since the last state the patient transitioned to) at the end of the study period or loss to follow-up. That is, the exact state is unknown but known to be in a certain set at the end of the study or loss to follow-up. We incorporated censoring (at the state level) in our model building for patients without known (observed) exact disease state at the end of our study period or loss to follow-up.

### Variable selection and model building

We selected demographic, clinical variables, and comorbidities that were available through the EHR as well as socioeconomic data from the ADI. We had a total of 59 variables in our dataset (Supplement Table 1). Due to the high dimensionality of our data, we needed to reduce the number of variables that might be highly correlated to avoid overfitting our model with redundant data. We ran several iterations of multivariate models using variable screening with our variables. Furthermore, we ran a version of multivariate multistate models without death transitions to determine if the effects of the variables for other state transitions remained the same. The Akaike information criterion (AIC) of each model was computed. AIC provides a good estimate for the overall model performance [27]. We selected our final multistate model based on the lowest AIC. Additionally, we performed a likelihood ratio test between the nested multistate models [28]. We validated our final model by comparing the diagnostic plots of observed versus expected prevalence estimates at a series of time points [23,25,29].

Finally, to verify that excluding the patients for whom both micro- and macrovascular complications were coded at the same visit did not result in biased results we did a sensitivity analysis and re-ran all the models by either assigning all these patients to the microvascular state first or assigning them to the macrovascular state first.

The data were analyzed using the multi-state modeling packaged from *R* statistical software version 4.0.2 [25].

## Results

### Study population

**Figure 1.**
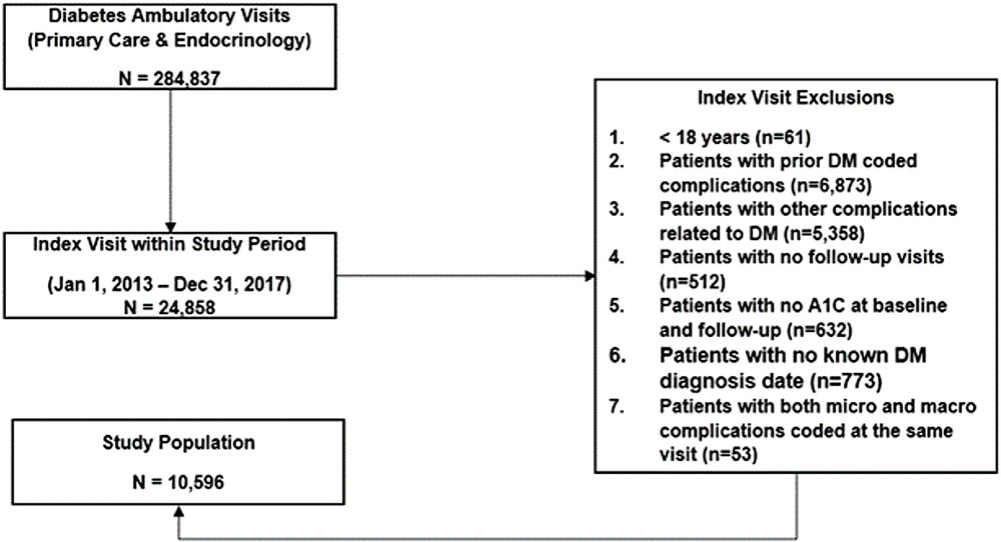
Flowchart of patient selection. A total of 10,596 patients were selected for our study population. DM = diabetes mellitus.

Our study population included 10,596 unique patients. Figure 1 illustrates the flowchart of patient selection for our study. Of the 10,596 patients, 56.2% were female, 64.3% were White, 28.4% were Black, 65.8% had commercial insurance, 28.6% were on Medicare, 1.9% were on Medicaid or dual insurance, and 0.8% were categorized as self-pay. Mean age (standard deviation) was 54.7 (14.1) years. A large majority of patients had type 2 diabetes (93.1%) and 1.8% have had diabetes for less than 5 years, 92.4% for 5 to 10 years, and 5.9% for more than 10 years. Many patients were former/current smokers (42.8%). The median (interquartile range) follow-up was 4.72 (3.11-6.55) years. The range of follow-up for our study population was 1.17 years to 6.99 years.

### Variable selection

**Figure 2.**
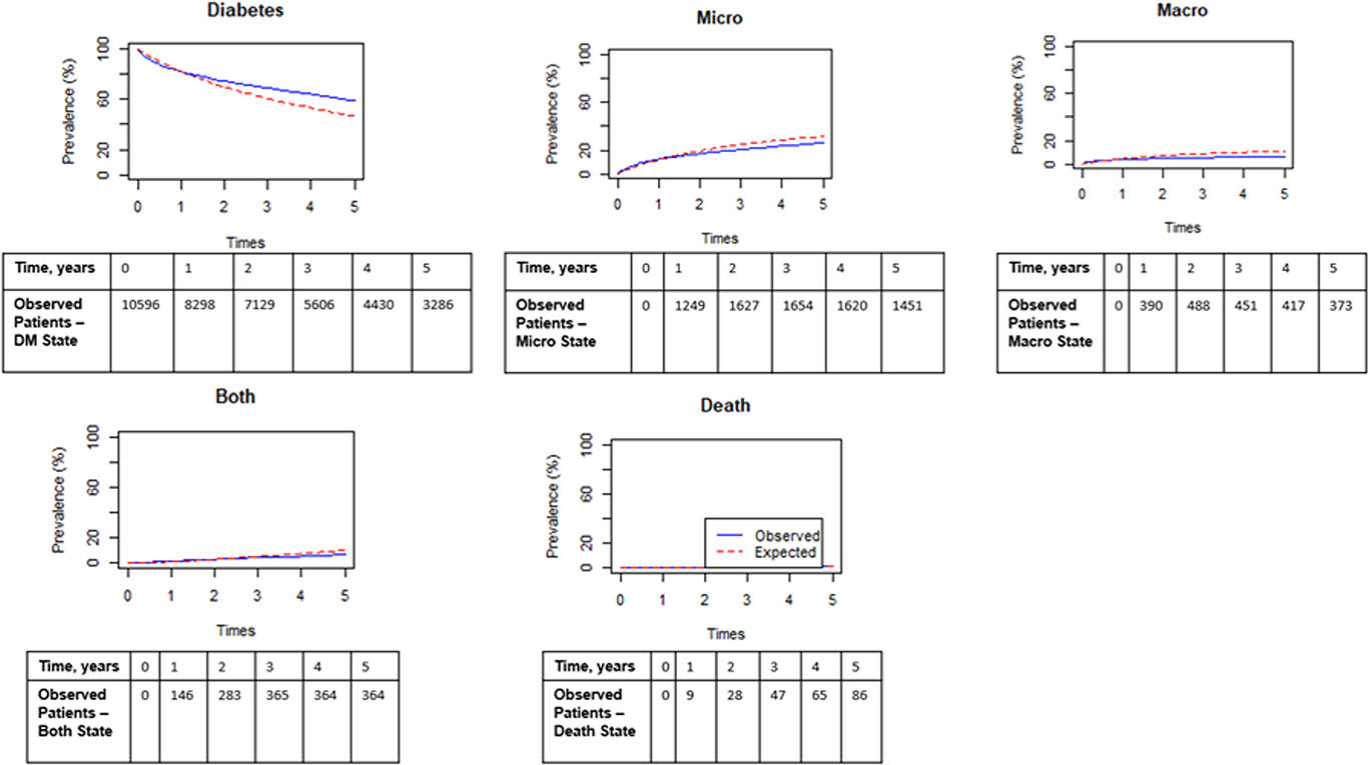
Model diagnostic plot of final model, observed vs expected (estimated) patients for each state over time. Tables of observed number of patients for each state.

We started with a null model that included all 59 variables for each transition for our first iteration multistate model. We then performed variable screening to drop covariates that were not significant. We stopped the iterative process when the AIC difference between two nested models was small and not decreasing. Our initial null model had an AIC of 42,017.42 and our final multistate model had an AIC of 38,061.59. The AIC results indicated that a simpler model containing fewer variables was more favorable than a model that included all the variables. And lastly, our diagnostic plots show the prevalence of the observed vs expected states (Figure 2). The diagnostic plots from each iteration of the multivariate multistate model were very similar, which reinforces that a simpler model can be favorable to avoid overfitting and achieve better interpretability.

The variables chosen for our final model are listed for each transition in Table 1. The transition from the DM State to the Microvascular State included 32 unique covariates – 6 demographics, 3 vitals, 12 clinical labs, 10 comorbidities, and ADI. DM State to Macrovascular State had 21 covariates – 4 demographics, 2 vitals, 6 clinical labs, and 9 comorbidities. DM State to Death State had no significant variables during our iterative process therefore there were no variables for our final model. Microvascular State to Both State had 20 covariates – 4 demographics, 1 vital, 5 clinical labs, and 10 comorbidities. Microvascular State to Death State had no significant variables. Macrovascular State to Both State had 16 covariates – 4 demographics, 1 vital, 5 clinical labs, and 6 comorbidities. Macrovascular State to Death State included only one clinical lab. The transition from Both to Death State had 5 significant variables, which included 1 demographic and 4 clinical lab variables. The models for the transitions that had no significant variables did not converge because of the low number of observations.

**Table 1.** Final multistate model; significant covariates for each transition and their hazard ratios [95% CI]

	Covariates	Diabetes →Microvascular	Diabetes →Macrovascular	Microvascular →Both	Macrovascular →Both	Macrovascular →Death	Both →Death
Demographics	Age	1.023 [1.019,1.027]	1.049 [1.043,1.056]	1.047 [1.038,1.056]	1.030 [1.017,1.042]		1.046 [1.023,1.068]
	Female vs Male	0.910 [0.838,0.987]	0.817 [0.723,0.923]	0.672 [0.568,0.796]			
	Black vs White						
	Other Race vs White				1.512 [1.000,2.314]		
	Non-Hispanic vs Hispanic						
	Medicare vs Commercial	1.549 [1.416,1.694]	1.315 [1.143,1.512]				
	Medicaid/Dual vs Commercial	1.485 [1.136,1.943]	2.874 [1.989,4.152]	2.043 [1.510,2.764]	1.966 [1.363,2.836]		
	Self Pay vs Commercial						
	Former/Current vs Never Smoker	1.183 [1.101,1.270]	1.408 [1.259,1.574]	1.315 [1.125,1.537]	1.327 [1.092,1.613]		
	Type 2 vs Type 1	0.616 [0.523,0.726]					
DOD	<5 vs 10+ years DOD	0.735 [0.586,0.922]					
	<10 vs 10+ years DOD	0.699 [0.538,0.908]					
Vitals	BMI < 18.5 vs BMI < 25						
	BMI < 30.0 vs BMI < 25						
	BMI > 30.0 vs BMI < 25						
	Blood Pressure Systolic	1.010 [1.007,1.013]	1.008 [1.004,1.012]	1.009 [1.004,1.014]	1.012 [1.006,1.018]		
	Blood Pressure Diastolic	0.989 [0.985,0.994]	0.990 [0.982,0.997]				
	Heart Rate	1.007 [1.004,1.009]					
Clinical Labs	Alkaline Phosphatase	1.002 [1.001,1.003]			1.005 [1.002,1.007]		
	Alanine Aminotransferase						
	Albumin	0.724 [0.643,0.814]	0.819 [0.687,0.977]	0.664 [0.563,0.784]			0.474 [0.323,0.696]
	Anion Gap	1.010 [1.000,1.024]					
	Aspartate Aminotransferase	1.002 [1.000,1.003]		1.005 [1.002,1.008]			
	Bilirubin	1.106 [1.025, 1.194]					1.425 [1.172,1.732]
	Glucose	1.001 [1.000,1.001]					
	Hemoglobin	0.937 [0.911,0.964]		0.909 [0.863,0.957]	0.868 [0.818,0.922]		
	Hemoglobin A1c	1.100 [1.074,1.126]	1.051 [1.019,1.084]	1.151 [1.105,1.199]	1.161 [1.103,1.222]		
	Urea Nitrogen	1.057 [1.050,1.064]	1.021 [1.009,1.032]		1.049 [1.034,1.065]		
	Low Calcium vs Normal Calcium						
	High Calcium vs Normal Calcium	1.230 [1.077,1.405]					
	Carbon Dioxide						
	Cholesterol						
	High Density Lipoprotein		0.994 [0.990,0.998]				
	Platelet						
	Low Potassium vs Normal Potassium						
	High Potassium vs Normal Potassium						
	Protein	1.198 [1.100,1.304]			0.745 [0.622,0.892]		
	Low Sodium vs Normal Sodium	1.409 [1.174,1.692]	1.408 [1.036,1.913]	1.541 [1.136,2.091]			
	High Sodium vs Normal Sodium	1.380 [1.144,1.664]	1.441 [1.086,1.912]				
	Thyroid Stimulating Hormone		1.015 [1.000,1.037]				1.072 [1.020,1.137]
	Triglycerides						
	White Blood Cell					1.272 [1.131,1.430]	1.061 [1.000,1.130]
Elixhauser Comorbidity	AIDS/HIV						
	Alcohol Abuse						
	Anemia Deficiency	1.174 [1.015,1.359]		1.364 [1.074,1.732]			
	Cardiac Arrhythmias		1.342 [1.157,1.556]				
	Chronic Pulmonary Disease	1.221 [1.121,1.329]	1.338 [1.177,1.522]	1.273 [1.075,1.506]			
	Coagulopathy	0.811 [0.668,0.984]		1.388 [1.043,1.848]			
	Congestive Heart Failure		2.053 [1.559,2.704]	1.954 [1.354,2.819]	1.733 [1.112,1.695]		
	Depression		1.357 [1.195,1.541]	1.270 [1.069,1.508]	1.373 [1.112,1.695]		
	Drug Abuse						
	Fluid and Electrolyte Disorders	1.230 [1.117,1.354]					
	Hypertension	1.489 [1.117,1.354]	1.205 [1.039,1.398]		1.606 [1.119,2.306]		
	Hypothyroidism	1.104 [1.014,1.202]					
	Liver Disease	1.277 [1.148,1.420]	1.250 [1.064,1.468]	1.267 [1.043,1.540]			
	Lymphoma						
	Obesity						
	Other Neurological Disorders	1.273 [1.085,1.493]		1.377 [1.053,1.801]	1.544 [1.091,2.184]		
	Paralysis		1.692 [1.103,2.596]				
	Peripheral Vascular Disorders		1.561 [1.229,1.984]		1.752 [1.412,2.175]		
	Psychoses						
	Pulmonary Circulation Disorders	1.412 [1.128,1.768]					
	Renal Failure	3.703 [2.986,4.592]		1.556 [1.188,2.038]			
	Rheumatoid Arthritis			1.469 [1.126,1.917]			
	Tumor/Metastatic Cancer				1.318 [1.029,1.689]		
	Valvular Disease		1.488 [1.212,1.825]	1.366 [1.081,1.726]			
	Weight Loss						
ADI	3–4 vs 1–2	1.211 [1.085,1.350]					
	5–6 vs 1–2	1.174 [1.052,1.310]					
	7–8 vs 1–2	1.229 [1.100,1.373]					
	9–10 vs 1–2	1.365 [1.217,1.532]					

CI = confidence interval; DOD = duration of diabetes; ADI = area deprivation index.

Table 1 shows the HR of each covariate for all transitions. For example, HRs for age, Medicaid/Dual insurance, smoking status, systolic blood pressure, and HbA1c are significant across all transitions to Microvascular, Macrovascular, and Both States. Women are less likely to develop microvascular and macrovascular complications than men (HR = 0.910 [0.838, 0.987]), as well as less likely to develop an additional macrovascular complication if they already had a microvascular complication (HR = 0.672 [0.568–0.796]). Patients of “Other Races” vs White are more likely to develop a microvascular complication with an existing macrovascular complication (HR = 1.512 [1.000, 2.314]). ADI is significant for patients who develop microvascular complications. Individuals who reside in the most disadvantaged neighborhoods (9–10) have an HR of 1.365 [1.217, 1.532] compared to those who reside in the least disadvantaged neighborhoods (1–2).

### Multistate model

**Figure 3.**
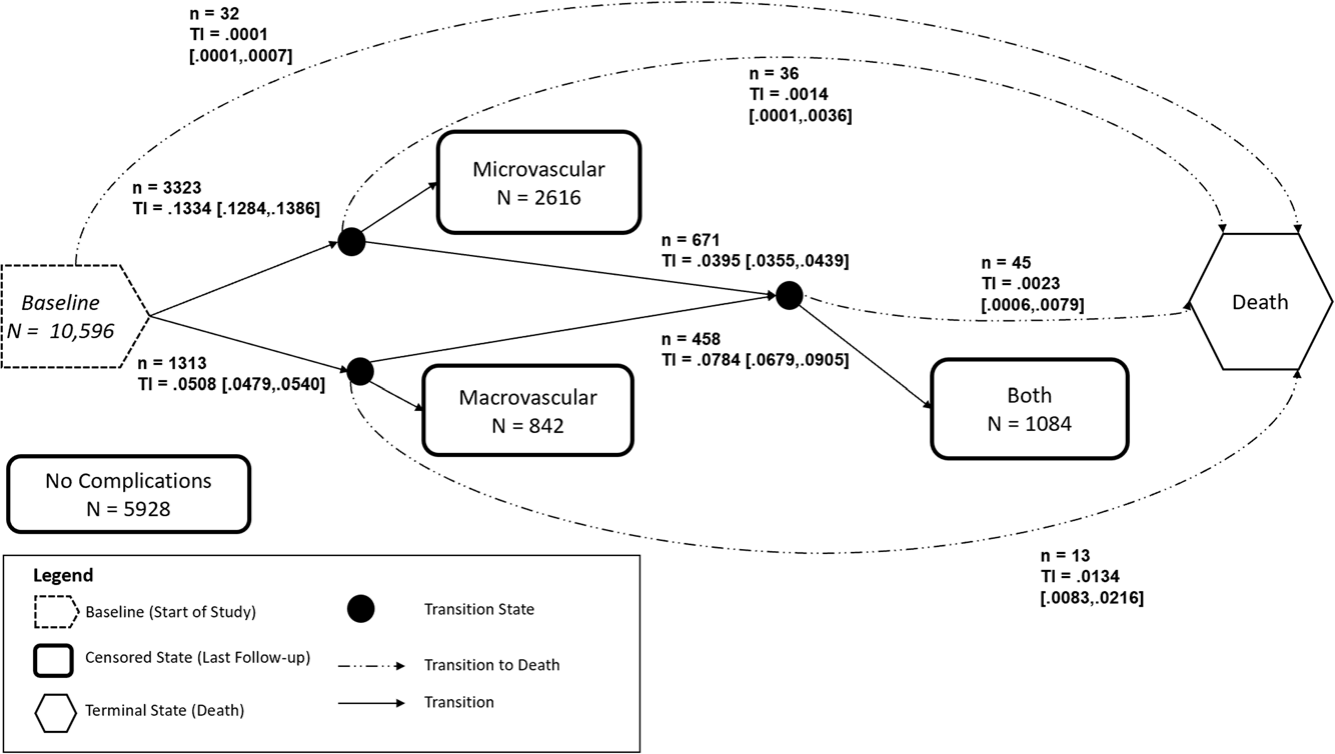
Five-state model for examining the progression of diabetes-related complications using electronic health records among diabetes patients. N, number of censored patients at the end of follow-up or loss to follow-up; n, number of observed transitions; TI [], transition intensity [95% CI]. Transition from diabetes state to Both State is not an allowable transition. Both State refers to patients who have a micro- and macrovascular complication. Death is the final absorbing state.

The number of patients moving from one state to the other is described in Figure 3. A total of 5928 patients did not develop any complications by the end of our study period, 32 died with no reported complications, 3323 developed microvascular complications, and 1313 developed macrovascular complications. Of the 3323 who developed microvascular complications, 671 further developed macrovascular complications and 36 died. Of the 1313 who developed macrovascular complications, 458 also developed microvascular complications and 13 died. There were 1129 who developed both a microvascular and macrovascular complication and 45 from that group died.

From our 5-state model, we estimated the transition intensity matrix, which provides the instantaneous rate of moving from one state to another state. Figure 3 shows that a patient has a faster rate of developing a microvascular complication compared to a macrovascular complication, 0.1334 [0.1284, .1386] versus 0.0508 [0.0479, .0540]. However, a patient has a higher instantaneous rate of developing a second complication if they already had a macrovascular complication compared to a microvascular complication, 0.0784 [0.0679, .0905] versus 0.0395 [0.0355, .0439].

From our model we also calculated the one-, three-, and five-year transition probabilities, providing the risk over time of getting to a particular state (Table 2). As time increases, the probability of staying in a DM State with no complications decreased from 83.2% [82.7%, 83.6%], to 57.5% [56.5%, 58.5%], and 39.8% [38.7%, 41.0%] at one-, three-, and five-year respectively. The risk of developing microvascular complications increased over time, with probabilities of 11.9% [11.5%, 12.4%], 28.8% [27.8%, 29.6%], and 38.8% [37.7%, 39.9%]. The risk over time to transition from no complications to a macrovascular complication increases from 4.4% [4.2%, 4.7%], to 10.3% [9.7%, 10.9%] and 13.3% [12.4%, 14.0%]. There is a smaller risk of developing a macrovascular complication compared to a microvascular complication because of a lower instantaneous rate as described in Figure 3. Among patients who transition to the Both State, patients with an existing macrovascular complication will have double the probability of getting a microvascular complication (7.5% [6.5%, 8.6%]) compared to those with an existing microvascular complication (3.8% [3.5%, 4.2%]) at one-year and this trend continues at three- and five-year.

**Table 2.** Estimated 1-year, 3-year, and 5-year state-to-state transition probabilities among diabetes patients

Year and State	Maximum Likelihood Estimate (95% CI)				
	Diabetes	Microvascular	Macrovascular	Both	Death
1 year					
Diabetes	0.832 (0.827,0.836)	0.119 (0.115,0.124)	0.044 (0.042,0.047)	0.004 (0.003,0.005)	0.000 (0.000,0.001)
Microvascular	–	0.960 (0.955,0.964)	–	0.038 (0.035,0.042)	0.002 (0.001,0.004)
Macrovascular	–	–	0.923 (0.910,0.933)	0.075 (0.065,0.086)	0.003 (0.001,0.008)
Both	–	–	–	0.987 (0.978,0.992)	0.013 (0.008,0.022)
Death	–	–	–	–	1.0000
3 years					
Diabetes	0.575 (0.565,0.585)	0.288 (0.278,0.296)	0.103 (0.097,0.109)	0.032 (0.030,0.035)	0.002 (0.001,0.003)
Microvascular	–	0.885 (0.872,0.896)	–	0.109 (0.099,0.121)	0.006 (0.004,0.012)
Macrovascular	–	–	0.785 (0.754,0.810)	0.205 (0.179,0.233)	0.10 (0.006,0.024)
Both	–	–	–	0.961 (0.938,0.976)	0.039 (0.024,0.062)
Death	–	–	–	–	1.0000
5 years					
Diabetes	0.398 (0.387,0.410)	0.388 (0.377,0.399)	0.133 (0.124,0.140)	0.077 (0.071,0.083)	0.005 (0.004,0.008)
Microvascular	–	0.815 (0.796,0.830)	–	0.172 (0.157,0.190)	0.012 (0.008,0.021)
Macrovascular	–	–	0.668 (0.623,0.701)	0.311 (0.277,0.348)	0.021 (0.013,0.043)
Both	–	–	–	0.935 (0.896,0.959)	0.065 (0.041,0.104)
Death	–	–	–	–	1.0000

An example of how to interpret the table: In 1 year, a patient will stay in a DM State (no complications) with a probability of 83.2%, transition from DM State to Microvascular State with a probability of 11.9%, transition from DM to Macrovascular State with a probability of 4.4% and have a 0.4% of transitioning from a DM State to a Death State. For patients with an existing microvascular complication, they have a 96.0% probability of staying in the Microvascular State, 3.8% probability of developing a macrovascular complication and transitioning to the Both State, and 0.2% of transition from the Microvascular State to Death State. For those that have an existing macrovascular complication, they have a 92.3% probability of staying in that state, a 7.5% of developing a microvascular complication and transitioning to the Both State, and a 0.3% of transitioning from the Macrovascular State to Death State. Those that are in the Both State (having a micro- and macrovascular complication) have a 98.7% probability of staying in that state and a 1.3% probability of transitioning to the Death State.

CI = confidence interval, DM = diabetes mellitus.

The sensitivity analysis did not show any differences in the results whether all the 53 patients who transitioned directly from diabetes to Both State at the same visit were assigned to the Diabetes to Micro first (all micro) or to the Diabetes to Macro first (all macro) as shown in Supplement Tables 3 and 4. The fact that the results of the 3 models were very similar corroborated our decision to exclude the 53 patients who transitioned to both from the diabetes state as mentioned in our study population paragraph and in Figure 1.

## Discussion

The multistate model we developed shows the rate and risk of a patient transitioning from having no complication to having multiple complications. The results show that patients had a higher rate of developing microvascular complications compared to macrovascular complications. Incorporating variables from multisource datasets allowed us to explore the effects of covariates on each transition. For example, women seem to be less likely to develop macrovascular complications, which is unexpected considering the results of multiple studies showing a higher risk for cardiovascular complications in diabetic women compared to diabetic men [30–32]. However, many of these studies report separately the relative risk of cardiovascular complications in diabetic versus nondiabetic men and women. Women may have a higher relative risk than men because the cardiovascular burden in nondiabetic women is lower than the cardiovascular burden in nondiabetic men. In our study, we compared directly diabetic women to diabetic men and excluded from our population individuals with known cardiovascular disease at baseline. On the other hand, all the complications in our database are defined by diagnosis codes entered during or after a clinical encounter. It is possible that cardiovascular complications diagnoses were missed in women because of atypical symptoms frequently occurring in diabetic women [30]. We also found that women were least likely to develop microvascular complications, which is consistent with studies showing men with a higher risk of developing microalbuminuria and diabetic retinopathy [33–35].

Our model shows that an increase in HbA1c is significant for the development of both micro- and macrovascular complications, which emphasizes the importance of controlling HbA1c. The effects of HbA1c and systolic blood pressure levels are consistent with results found in other studies that applied multistate modeling to microvascular complications [11–13]. Not surprisingly, smoking is a risk factor for developing a new complication at any step of the model. ADI, which is not included in EHRs, is a significant factor for patients who develop microvascular complications, with patients who reside in the most disadvantaged neighborhoods having a higher likelihood of developing a complication compared to those who reside in the least disadvantaged neighborhood. Low socioeconomic status has been shown to be associated with diabetic complications in analyses using logistic or Cox proportional hazard regressions and the prevalence of obesity and diabetes has been shown to be associated with neighborhood deprivation but indicators of socioeconomic status or ADI levels have not been included in previous diabetes-related multistate models [36–39].

Our study considered all complications associated with DM by including diagnosis codes specifically for DM and those related to DM. Most applications of multistate modeling limit their transitions to only include a subset of complications associated with DM with a relatively small number of covariates.

Although our model shows predictive strength it also has several limitations. The first is that we used diagnosis codes to identify complications, which may not reflect the exact timing of occurrence of a complication and may also miss some complications. However, by broadening our outcomes to not only ICD9/10 DM specific codes but also to ICD 9/10 and CPT codes related to DM, we were able to capture more patients who experienced complications who normally would have been missed as transitioning to a Micro- or Macrovascular State. Second, our model was not validated with an external dataset to determine performance. Nevertheless, we examined model selection criteria such as AIC at each iteration of our model building. Furthermore, our diagnostic plots show validity in our analysis. Since we did not have medication in our data, we based DM control on HbA1c. Also, by categorizing our complications as either micro- or macrovascular, we are assuming that the disease process is similar for each complication within the microvascular or macrovascular categories. This might be reflected by the high number of variables selected for the transition from DM State to Microvascular State. Lastly, since the sample size for transitions to death was small, this might not precisely infer the results of significant variables in the death transitions. However, we ran separate iterations of the multistate model without the death transitions included and it did not affect our estimations for other transitions.

A multistate modeling approach to DM complications can help understand the progression of DM complications which will not only aid a physician’s ability to provide better care but also anticipate complications and plan interventions to reduce the patient’s risk of an event. The burden that DM presents in the long term not only affects the patients’ health but also their life expectancy. With our model, a physician could, for example, vary HbA1C levels and show a patient what is the risk of developing microvascular complications at one-, five-, and ten-years according to their personal characteristics.

Future work will focus on validating the multistate model with an external dataset of DM patients to test the strength of the model’s predictive capabilities. Once validated, the algorithm could be integrated into EHR to allow physicians to predict long-term risk according to a patient’s characteristics.

## Supporting information

Li et al. supplementary material Li et al. supplementary material

## Supplementary material

The supplementary material for this article can be found at https://doi.org/10.1017/cts.2024.583.
