# Supplementary material for: Building a multistate model from electronic health records data for modeling long-term diabetes complications
Source: J Clin Transl Sci. 2024 Sep 23;8(1):e133. doi: 10.1017/cts.2024.583 (PMC11428072; doi:10.1017/cts.2024.583)
Supplement: Li et al. supplementary material [file S2059866124005831sup001.docx]

**Supplement Tables**

Table 1. Categories of variables included in our study of diabetic patients.

| **Category** | **Variables** |
| --- | --- |
| Demographic | Age  Gender  Race  Ethnicity  Insurance  Smoking Status  Diabetes Type  Duration of Diabetes |
| Vitals | Body Mass Index  Blood Pressure Systolic  Blood Pressure Diastolic  Heart Rate |
| Clinical Labs | Alanine Aminotransferase Serum  Albumin Serum  Alkaline Phosphatase Serum  Anion Gap Serum  Aspartate Aminotransferase Serum  Bilirubin Serum  Blood Glucose  Blood Hemoglobin  Blood Hemoglobin A1c  Blood Urea Nitrogen  Calcium Serum  Carbon Dioxide Total Serum  Cholesterol Serum  High Density Lipoprotein Serum  Platelet Count  Potassium Serum  Protein Total Serum  Sodium Serum  Thyroid Stimulating Hormone Serum  Triglycerides Serum  White Blood Cell Count |
| Elixhauser Comorbidity | History of AIDS/HIV  History of Alcohol abuse  History of Anemia deficiency  History of Cardiac arrhythmias  History of Chronic pulmonary disease  History of Coagulopathy  History of Congestive heart failure  History of Depression  History of Drug abuse  History of Fluid and electrolyte disorders  History of Hypertension  History of Hypothyroidism  History of Liver Disease  History of Lymphoma  History of Obesity  History of Other neurological disorders  History of Paralysis  History of Peripheral vascular disorders  History of Psychoses  History of Pulmonary circulation disorders  History of Renal failure  History of Rheumatoid arthritis  History of Tumor or Metastatic cancer  History of Valvular disease  History of Weight loss |
| Area Deprivation Index | 1-10, ranked neighborhood score |

Table 2. ICD9/10 diagnosis codes and CPT codes for DM complications and DM related complications. Categorized into microvascular and macrovascular complications.

| **Microvascular Complications** | | | **Macrovascular Complications** | | |
| --- | --- | --- | --- | --- | --- |
| DM-specific  Complications | DM-related Complications | CPT Complications | DM-specific  Complications | DM-related Complications | CPT Complications |
| \| E10.2 \| ICD10 \| \| --- \| --- \| \| E10.21 \| ICD10 \| \| E10.22 \| ICD10 \| \| E10.29 \| ICD10 \| \| E11.2 \| ICD10 \| \| E11.21 \| ICD10 \| \| E11.22 \| ICD10 \| \| E11.29 \| ICD10 \| \| E13.2 \| ICD10 \| \| E13.21 \| ICD10 \| \| E13.22 \| ICD10 \| \| E13.29 \| ICD10 \| \| 250.4 \| ICD9 \| \| 250.41 \| ICD9 \| \| 250.42 \| ICD9 \| \| 250.43 \| ICD9 \| \| 249.4 \| ICD9 \| \| 249.40 \| ICD9 \| \| 249.41 \| ICD9 \| \| E08.2 \| ICD10 \| \| E08.21 \| ICD10 \| \| E08.22 \| ICD10 \| \| E08.29 \| ICD10 \| \| E10.4 \| ICD10 \| \| E10.40 \| ICD10 \| \| E10.41 \| ICD10 \| \| E10.42 \| ICD10 \| \| E10.43 \| ICD10 \| \| E10.44 \| ICD10 \| \| E10.49 \| ICD10 \| \| E10.61 \| ICD10 \| \| E10.610 \| ICD10 \| \| E10.618 \| ICD10 \| \| E10.621 \| ICD10 \| \| E11.4 \| ICD10 \| \| E11.40 \| ICD10 \| \| E11.41 \| ICD10 \| \| E11.42 \| ICD10 \| \| E11.43 \| ICD10 \| \| E11.44 \| ICD10 \| \| E11.49 \| ICD10 \| \| E11.61 \| ICD10 \| \| E11.610 \| ICD10 \| \| E11.618 \| ICD10 \| \| E11.621 \| ICD10 \| \| E13.4 \| ICD10 \| \| E13.40 \| ICD10 \| \| E13.41 \| ICD10 \| \| E13.42 \| ICD10 \| \| E13.43 \| ICD10 \| \| E13.44 \| ICD10 \| \| E13.49 \| ICD10 \| \| E13.61 \| ICD10 \| \| E13.610 \| ICD10 \| \| E13.618 \| ICD10 \| \| E13.621 \| ICD10 \| \| 250.6 \| ICD9 \| \| 250.61 \| ICD9 \| \| 250.62 \| ICD9 \| \| 250.63 \| ICD9 \| \| 249.6 \| ICD9 \| \| 249.60 \| ICD9 \| \| 249.61 \| ICD9 \| \| 357.2 \| ICD9 \| \| E08.4 \| ICD10 \| \| E08.40 \| ICD10 \| \| E08.41 \| ICD10 \| \| E08.42 \| ICD10 \| \| E08.43 \| ICD10 \| \| E08.44 \| ICD10 \| \| E08.49 \| ICD10 \| \| E08.61 \| ICD10 \| \| E08.610 \| ICD10 \| \| E08.618 \| ICD10 \| \| E08.621 \| ICD10 \| \| E10.3 \| ICD10 \| \| E10.31 \| ICD10 \| \| E10.311 \| ICD10 \| \| E10.319 \| ICD10 \| \| E10.32 \| ICD10 \| \| E10.321 \| ICD10 \| \| E10.3211 \| ICD10 \| \| E10.3212 \| ICD10 \| \| E10.3213 \| ICD10 \| \| E10.3219 \| ICD10 \| \| E10.329 \| ICD10 \| \| E10.3291 \| ICD10 \| \| E10.3292 \| ICD10 \| \| E10.3293 \| ICD10 \| \| E10.3299 \| ICD10 \| \| E10.33 \| ICD10 \| \| E10.331 \| ICD10 \| \| E10.3311 \| ICD10 \| \| E10.3312 \| ICD10 \| \| E10.3313 \| ICD10 \| \| E10.3319 \| ICD10 \| \| E10.339 \| ICD10 \| \| E10.3391 \| ICD10 \| \| E10.3392 \| ICD10 \| \| E10.3393 \| ICD10 \| \| E10.3399 \| ICD10 \| \| E10.34 \| ICD10 \| \| E10.341 \| ICD10 \| \| E10.3411 \| ICD10 \| \| E10.3412 \| ICD10 \| \| E10.3413 \| ICD10 \| \| E10.3419 \| ICD10 \| \| E10.349 \| ICD10 \| \| E10.3491 \| ICD10 \| \| E10.3492 \| ICD10 \| \| E10.3493 \| ICD10 \| \| E10.3499 \| ICD10 \| \| E10.35 \| ICD10 \| \| E10.351 \| ICD10 \| \| E10.3511 \| ICD10 \| \| E10.3512 \| ICD10 \| \| E10.3513 \| ICD10 \| \| E10.3519 \| ICD10 \| \| E10.352 \| ICD10 \| \| E10.3521 \| ICD10 \| \| E10.3522 \| ICD10 \| \| E10.3523 \| ICD10 \| \| E10.3529 \| ICD10 \| \| E10.353 \| ICD10 \| \| E10.3531 \| ICD10 \| \| E10.3532 \| ICD10 \| \| E10.3533 \| ICD10 \| \| E10.3539 \| ICD10 \| \| E10.354 \| ICD10 \| \| E10.3541 \| ICD10 \| \| E10.3542 \| ICD10 \| \| E10.3543 \| ICD10 \| \| E10.3549 \| ICD10 \| \| E10.355 \| ICD10 \| \| E10.3551 \| ICD10 \| \| E10.3552 \| ICD10 \| \| E10.3553 \| ICD10 \| \| E10.3559 \| ICD10 \| \| E10.359 \| ICD10 \| \| E10.3591 \| ICD10 \| \| E10.3592 \| ICD10 \| \| E10.3593 \| ICD10 \| \| E10.3599 \| ICD10 \| \| E10.37 \| ICD10 \| \| E10.37X1 \| ICD10 \| \| E10.37X2 \| ICD10 \| \| E10.37X3 \| ICD10 \| \| E10.37X9 \| ICD10 \| \| E10.39 \| ICD10 \| \| E11.3 \| ICD10 \| \| E11.31 \| ICD10 \| \| E11.311 \| ICD10 \| \| E11.319 \| ICD10 \| \| E11.32 \| ICD10 \| \| E11.321 \| ICD10 \| \| E11.3211 \| ICD10 \| \| E11.3212 \| ICD10 \| \| E11.3213 \| ICD10 \| \| E11.3219 \| ICD10 \| \| E11.329 \| ICD10 \| \| E11.3291 \| ICD10 \| \| E11.3292 \| ICD10 \| \| E11.3293 \| ICD10 \| \| E11.3299 \| ICD10 \| \| E11.33 \| ICD10 \| \| E11.331 \| ICD10 \| \| E11.3311 \| ICD10 \| \| E11.3312 \| ICD10 \| \| E11.3313 \| ICD10 \| \| E11.3319 \| ICD10 \| \| E11.339 \| ICD10 \| \| E11.3391 \| ICD10 \| \| E11.3392 \| ICD10 \| \| E11.3393 \| ICD10 \| \| E11.3399 \| ICD10 \| \| E11.34 \| ICD10 \| \| E11.341 \| ICD10 \| \| E11.3411 \| ICD10 \| \| E11.3412 \| ICD10 \| \| E11.3413 \| ICD10 \| \| E11.3419 \| ICD10 \| \| E11.349 \| ICD10 \| \| E11.3491 \| ICD10 \| \| E11.3492 \| ICD10 \| \| E11.3493 \| ICD10 \| \| E11.3499 \| ICD10 \| \| E11.35 \| ICD10 \| \| E11.351 \| ICD10 \| \| E11.3511 \| ICD10 \| \| E11.3512 \| ICD10 \| \| E11.3513 \| ICD10 \| \| E11.3519 \| ICD10 \| \| E11.352 \| ICD10 \| \| E11.3521 \| ICD10 \| \| E11.3522 \| ICD10 \| \| E11.3523 \| ICD10 \| \| E11.3529 \| ICD10 \| \| E11.353 \| ICD10 \| \| E11.3531 \| ICD10 \| \| E11.3532 \| ICD10 \| \| E11.3533 \| ICD10 \| \| E11.3539 \| ICD10 \| \| E11.354 \| ICD10 \| \| E11.3541 \| ICD10 \| \| E11.3542 \| ICD10 \| \| E11.3543 \| ICD10 \| \| E11.3549 \| ICD10 \| \| E11.355 \| ICD10 \| \| E11.3551 \| ICD10 \| \| E11.3552 \| ICD10 \| \| E11.3553 \| ICD10 \| \| E11.3559 \| ICD10 \| \| E11.359 \| ICD10 \| \| E11.3591 \| ICD10 \| \| E11.3592 \| ICD10 \| \| E11.3593 \| ICD10 \| \| E11.3599 \| ICD10 \| \| E11.37 \| ICD10 \| \| E11.37X1 \| ICD10 \| \| E11.37X2 \| ICD10 \| \| E11.37X3 \| ICD10 \| \| E11.37X9 \| ICD10 \| \| E11.39 \| ICD10 \| \| E13.3 \| ICD10 \| \| E13.31 \| ICD10 \| \| E13.311 \| ICD10 \| \| E13.319 \| ICD10 \| \| E13.32 \| ICD10 \| \| E13.321 \| ICD10 \| \| E13.3211 \| ICD10 \| \| E13.3212 \| ICD10 \| \| E13.3213 \| ICD10 \| \| E13.3219 \| ICD10 \| \| E13.329 \| ICD10 \| \| E13.3291 \| ICD10 \| \| E13.3292 \| ICD10 \| \| E13.3293 \| ICD10 \| \| E13.3299 \| ICD10 \| \| E13.33 \| ICD10 \| \| E13.331 \| ICD10 \| \| E13.3311 \| ICD10 \| \| E13.3312 \| ICD10 \| \| E13.3313 \| ICD10 \| \| E13.3319 \| ICD10 \| \| E13.339 \| ICD10 \| \| E13.3391 \| ICD10 \| \| E13.3392 \| ICD10 \| \| E13.3393 \| ICD10 \| \| E13.3399 \| ICD10 \| \| E13.34 \| ICD10 \| \| E13.341 \| ICD10 \| \| E13.3411 \| ICD10 \| \| E13.3412 \| ICD10 \| \| E13.3413 \| ICD10 \| \| E13.3419 \| ICD10 \| \| E13.349 \| ICD10 \| \| E13.3491 \| ICD10 \| \| E13.3492 \| ICD10 \| \| E13.3493 \| ICD10 \| \| E13.3499 \| ICD10 \| \| E13.35 \| ICD10 \| \| E13.351 \| ICD10 \| \| E13.3511 \| ICD10 \| \| E13.3512 \| ICD10 \| \| E13.3513 \| ICD10 \| \| E13.3519 \| ICD10 \| \| E13.352 \| ICD10 \| \| E13.3521 \| ICD10 \| \| E13.3522 \| ICD10 \| \| E13.3523 \| ICD10 \| \| E13.3529 \| ICD10 \| \| E13.353 \| ICD10 \| \| E13.3531 \| ICD10 \| \| E13.3532 \| ICD10 \| \| E13.3533 \| ICD10 \| \| E13.3539 \| ICD10 \| \| E13.354 \| ICD10 \| \| E13.3541 \| ICD10 \| \| E13.3542 \| ICD10 \| \| E13.3543 \| ICD10 \| \| E13.3549 \| ICD10 \| \| E13.355 \| ICD10 \| \| E13.3551 \| ICD10 \| \| E13.3552 \| ICD10 \| \| E13.3553 \| ICD10 \| \| E13.3559 \| ICD10 \| \| E13.359 \| ICD10 \| \| E13.3591 \| ICD10 \| \| E13.3592 \| ICD10 \| \| E13.3593 \| ICD10 \| \| E13.3599 \| ICD10 \| \| E13.37 \| ICD10 \| \| E13.37X1 \| ICD10 \| \| E13.37X2 \| ICD10 \| \| E13.37X3 \| ICD10 \| \| E13.37X9 \| ICD10 \| \| E13.39 \| ICD10 \| \| 250.5 \| ICD9 \| \| 250.51 \| ICD9 \| \| 250.52 \| ICD9 \| \| 250.53 \| ICD9 \| \| 249.5 \| ICD9 \| \| 249.50 \| ICD9 \| \| 249.51 \| ICD9 \| \| 362.01 \| ICD9 \| \| 362.02 \| ICD9 \| \| 362.03 \| ICD9 \| \| 362.04 \| ICD9 \| \| 362.05 \| ICD9 \| \| 362.06 \| ICD9 \| \| E08.3 \| ICD10 \| \| E08.31 \| ICD10 \| \| E08.311 \| ICD10 \| \| E08.319 \| ICD10 \| \| E08.32 \| ICD10 \| \| E08.321 \| ICD10 \| \| E08.3211 \| ICD10 \| \| E08.3212 \| ICD10 \| \| E08.3213 \| ICD10 \| \| E08.3219 \| ICD10 \| \| E08.329 \| ICD10 \| \| E08.3291 \| ICD10 \| \| E08.3292 \| ICD10 \| \| E08.3293 \| ICD10 \| \| E08.3299 \| ICD10 \| \| E08.33 \| ICD10 \| \| E08.331 \| ICD10 \| \| E08.3311 \| ICD10 \| \| E08.3312 \| ICD10 \| \| E08.3313 \| ICD10 \| \| E08.3319 \| ICD10 \| \| E08.339 \| ICD10 \| \| E08.3391 \| ICD10 \| \| E08.3392 \| ICD10 \| \| E08.3393 \| ICD10 \| \| E08.3399 \| ICD10 \| \| E08.34 \| ICD10 \| \| E08.341 \| ICD10 \| \| E08.3411 \| ICD10 \| \| E08.3412 \| ICD10 \| \| E08.3413 \| ICD10 \| \| E08.3419 \| ICD10 \| \| E08.349 \| ICD10 \| \| E08.3491 \| ICD10 \| \| E08.3492 \| ICD10 \| \| E08.3493 \| ICD10 \| \| E08.3499 \| ICD10 \| \| E08.35 \| ICD10 \| \| E08.351 \| ICD10 \| \| E08.3511 \| ICD10 \| \| E08.3512 \| ICD10 \| \| E08.3513 \| ICD10 \| \| E08.3519 \| ICD10 \| \| E08.352 \| ICD10 \| \| E08.3521 \| ICD10 \| \| E08.3522 \| ICD10 \| \| E08.3523 \| ICD10 \| \| E08.3529 \| ICD10 \| \| E08.353 \| ICD10 \| \| E08.3531 \| ICD10 \| \| E08.3532 \| ICD10 \| \| E08.3533 \| ICD10 \| \| E08.3539 \| ICD10 \| \| E08.354 \| ICD10 \| \| E08.3541 \| ICD10 \| \| E08.3542 \| ICD10 \| \| E08.3543 \| ICD10 \| \| E08.3549 \| ICD10 \| \| E08.355 \| ICD10 \| \| E08.3551 \| ICD10 \| \| E08.3552 \| ICD10 \| \| E08.3553 \| ICD10 \| \| E08.3559 \| ICD10 \| \| E08.359 \| ICD10 \| \| E08.3591 \| ICD10 \| \| E08.3592 \| ICD10 \| \| E08.3593 \| ICD10 \| \| E08.3599 \| ICD10 \| \| E08.37 \| ICD10 \| \| E08.37X1 \| ICD10 \| \| E08.37X2 \| ICD10 \| \| E08.37X3 \| ICD10 \| \| E08.37X9 \| ICD10 \| \| E08.39 \| ICD10 \| | \| 403.00 \| ICD9 \| \| --- \| --- \| \| 403.01 \| ICD9 \| \| 403.10 \| ICD9 \| \| 403.11 \| ICD9 \| \| 403.90 \| ICD9 \| \| 403.91 \| ICD9 \| \| 580.8 \| ICD9 \| \| 580.81 \| ICD9 \| \| 580.89 \| ICD9 \| \| 580.9 \| ICD9 \| \| 581.0 \| ICD9 \| \| 581.1 \| ICD9 \| \| 581.3 \| ICD9 \| \| 581.81 \| ICD9 \| \| 581.9 \| ICD9 \| \| 582.1 \| ICD9 \| \| 582.8 \| ICD9 \| \| 582.89 \| ICD9 \| \| 582.9 \| ICD9 \| \| 583.1 \| ICD9 \| \| 583.2 \| ICD9 \| \| 583.8 \| ICD9 \| \| 583.81 \| ICD9 \| \| 583.89 \| ICD9 \| \| 583.9 \| ICD9 \| \| 584 \| ICD9 \| \| 584.5 \| ICD9 \| \| 584.8 \| ICD9 \| \| 584.9 \| ICD9 \| \| 585 \| ICD9 \| \| 585.1 \| ICD9 \| \| 585.2 \| ICD9 \| \| 585.3 \| ICD9 \| \| 585.4 \| ICD9 \| \| 585.5 \| ICD9 \| \| 585.6 \| ICD9 \| \| 585.9 \| ICD9 \| \| 586 \| ICD9 \| \| 587 \| ICD9 \| \| 588.0 \| ICD9 \| \| 588.8 \| ICD9 \| \| 588.81 \| ICD9 \| \| 588.89 \| ICD9 \| \| 588.9 \| ICD9 \| \| 589.1 \| ICD9 \| \| 589.9 \| ICD9 \| \| I12.0 \| ICD10 \| \| N00.8 \| ICD10 \| \| N00.9 \| ICD10 \| \| N02.2 \| ICD10 \| \| N03.2 \| ICD10 \| \| N03.8 \| ICD10 \| \| N03.9 \| ICD10 \| \| N04.0 \| ICD10 \| \| N04.8 \| ICD10 \| \| N04.9 \| ICD10 \| \| N05.2 \| ICD10 \| \| N05.5 \| ICD10 \| \| N05.8 \| ICD10 \| \| N05.9 \| ICD10 \| \| N08 \| ICD10 \| \| N17.0 \| ICD10 \| \| N17.1 \| ICD10 \| \| N17.2 \| ICD10 \| \| N17.8 \| ICD10 \| \| N17.9 \| ICD10 \| \| N18.1 \| ICD10 \| \| N18.2 \| ICD10 \| \| N18.3 \| ICD10 \| \| N18.4 \| ICD10 \| \| N18.5 \| ICD10 \| \| N18.6 \| ICD10 \| \| N18.9 \| ICD10 \| \| N19 \| ICD10 \| \| N25.0 \| ICD10 \| \| N25.81 \| ICD10 \| \| N25.89 \| ICD10 \| \| N25.9 \| ICD10 \| \| N26.9 \| ICD10 \| \| 707.14 \| ICD9 \| \| 707.15 \| ICD9 \| \| L97.409 \| ICD10 \| \| L97.509 \| ICD10 \| \| 361.00 \| ICD9 \| \| 361.05 \| ICD9 \| \| 361.06 \| ICD9 \| \| 361.8 \| ICD9 \| \| 361.81 \| ICD9 \| \| 361.89 \| ICD9 \| \| 361.9 \| ICD9 \| \| 379.23 \| ICD9 \| \| 379.24 \| ICD9 \| \| H43.13 \| ICD10 \| | \| 67113 \| \| --- \| \| 67028 \| \| 67036 \| \| 14.31 \| \| 14.34 \| \| 14.35 \| \| 14.49 \| \| 14.54 \| \| 14.74 \| \| 14.75 \| \| 14.79 \| | \| E10.5 \| ICD10 \| \| --- \| --- \| \| E10.51 \| ICD10 \| \| E10.52 \| ICD10 \| \| E10.59 \| ICD10 \| \| E10.622 \| ICD10 \| \| E11.5 \| ICD10 \| \| E11.51 \| ICD10 \| \| E11.52 \| ICD10 \| \| E11.59 \| ICD10 \| \| E11.622 \| ICD10 \| \| E13.5 \| ICD10 \| \| E13.51 \| ICD10 \| \| E13.52 \| ICD10 \| \| E13.59 \| ICD10 \| \| E13.622 \| ICD10 \| \| 250.7 \| ICD9 \| \| 250.71 \| ICD9 \| \| 250.72 \| ICD9 \| \| 250.73 \| ICD9 \| \| 249.7 \| ICD9 \| \| 249.70 \| ICD9 \| \| 249.71 \| ICD9 \| \| E08.5 \| ICD10 \| \| E08.51 \| ICD10 \| \| E08.52 \| ICD10 \| \| E08.59 \| ICD10 \| \| E08.622 \| ICD10 \| | \| 404.00 \| ICD9 \|  \| Vascular \| \| --- \| --- \| --- \| --- \| \| 404.01 \| ICD9 \|  \| Vascular \| \| 404.10 \| ICD9 \|  \| Vascular \| \| 404.11 \| ICD9 \|  \| Vascular \| \| 404.13 \| ICD9 \|  \| Vascular \| \| 404.90 \| ICD9 \|  \| Vascular \| \| 404.91 \| ICD9 \|  \| Vascular \| \| 404.92 \| ICD9 \|  \| Vascular \| \| 404.93 \| ICD9 \|  \| Vascular \| \| 410 \| ICD9 \|  \| Vascular \| \| 410.00 \| ICD9 \|  \| Vascular \| \| 410.01 \| ICD9 \|  \| Vascular \| \| 410.10 \| ICD9 \|  \| Vascular \| \| 410.11 \| ICD9 \|  \| Vascular \| \| 410.12 \| ICD9 \|  \| Vascular \| \| 410.31 \| ICD9 \|  \| Vascular \| \| 410.40 \| ICD9 \|  \| Vascular \| \| 410.41 \| ICD9 \|  \| Vascular \| \| 410.42 \| ICD9 \|  \| Vascular \| \| 410.61 \| ICD9 \|  \| Vascular \| \| 410.70 \| ICD9 \|  \| Vascular \| \| 410.71 \| ICD9 \|  \| Vascular \| \| 410.72 \| ICD9 \|  \| Vascular \| \| 410.90 \| ICD9 \|  \| Vascular \| \| 410.91 \| ICD9 \|  \| Vascular \| \| 410.92 \| ICD9 \|  \| Vascular \| \| 411 \| ICD9 \|  \| Vascular \| \| 411.0 \| ICD9 \|  \| Vascular \| \| 411.1 \| ICD9 \|  \| Vascular \| \| 411.8 \| ICD9 \|  \| Vascular \| \| 411.89 \| ICD9 \|  \| Vascular \| \| 412 \| ICD9 \|  \| Vascular \| \| 413 \| ICD9 \|  \| Vascular \| \| 413.0 \| ICD9 \|  \| Vascular \| \| 413.9 \| ICD9 \|  \| Vascular \| \| 414 \| ICD9 \|  \| Vascular \| \| 414.00 \| ICD9 \|  \| Vascular \| \| 414.01 \| ICD9 \|  \| Vascular \| \| 414.02 \| ICD9 \|  \| Vascular \| \| 414.04 \| ICD9 \|  \| Vascular \| \| 414.05 \| ICD9 \|  \| Vascular \| \| 414.06 \| ICD9 \|  \| Vascular \| \| 414.07 \| ICD9 \|  \| Vascular \| \| 414.10 \| ICD9 \|  \| Vascular \| \| 414.11 \| ICD9 \|  \| Vascular \| \| 414.12 \| ICD9 \|  \| Vascular \| \| 414.2 \| ICD9 \|  \| Vascular \| \| 414.3 \| ICD9 \|  \| Vascular \| \| 414.4 \| ICD9 \|  \| Vascular \| \| 414.8 \| ICD9 \|  \| Vascular \| \| 414.9 \| ICD9 \|  \| Vascular \| \| 428 \| ICD9 \|  \| Vascular \| \| 428.0 \| ICD9 \|  \| Vascular \| \| 428.1 \| ICD9 \|  \| Vascular \| \| 428.2 \| ICD9 \|  \| Vascular \| \| 428.20 \| ICD9 \|  \| Vascular \| \| 428.21 \| ICD9 \|  \| Vascular \| \| 428.22 \| ICD9 \|  \| Vascular \| \| 428.23 \| ICD9 \|  \| Vascular \| \| 428.3 \| ICD9 \|  \| Vascular \| \| 428.30 \| ICD9 \|  \| Vascular \| \| 428.31 \| ICD9 \|  \| Vascular \| \| 428.32 \| ICD9 \|  \| Vascular \| \| 428.33 \| ICD9 \|  \| Vascular \| \| 428.40 \| ICD9 \|  \| Vascular \| \| 428.41 \| ICD9 \|  \| Vascular \| \| 428.42 \| ICD9 \|  \| Vascular \| \| 428.43 \| ICD9 \|  \| Vascular \| \| 428.9 \| ICD9 \|  \| Vascular \| \| 430 \| ICD9 \|  \| Vascular \| \| 431 \| ICD9 \|  \| Vascular \| \| 432.0 \| ICD9 \|  \| Vascular \| \| 432.1 \| ICD9 \|  \| Vascular \| \| 432.9 \| ICD9 \|  \| Vascular \| \| 433.0 \| ICD9 \|  \| Vascular \| \| 433.00 \| ICD9 \|  \| Vascular \| \| 433.01 \| ICD9 \|  \| Vascular \| \| 433.10 \| ICD9 \|  \| Vascular \| \| 433.11 \| ICD9 \|  \| Vascular \| \| 433.20 \| ICD9 \|  \| Vascular \| \| 433.21 \| ICD9 \|  \| Vascular \| \| 433.30 \| ICD9 \|  \| Vascular \| \| 433.31 \| ICD9 \|  \| Vascular \| \| 433.80 \| ICD9 \|  \| Vascular \| \| 433.9 \| ICD9 \|  \| Vascular \| \| 433.90 \| ICD9 \|  \| Vascular \| \| 434.01 \| ICD9 \|  \| Vascular \| \| 434.11 \| ICD9 \|  \| Vascular \| \| 434.90 \| ICD9 \|  \| Vascular \| \| 434.91 \| ICD9 \|  \| Vascular \| \| 435.0 \| ICD9 \|  \| Vascular \| \| 435.2 \| ICD9 \|  \| Vascular \| \| 435.3 \| ICD9 \|  \| Vascular \| \| 435.9 \| ICD9 \|  \| Vascular \| \| 436 \| ICD9 \|  \| Vascular \| \| 437.0 \| ICD9 \|  \| Vascular \| \| 437.1 \| ICD9 \|  \| Vascular \| \| 437.2 \| ICD9 \|  \| Vascular \| \| 437.3 \| ICD9 \|  \| Vascular \| \| 437.8 \| ICD9 \|  \| Vascular \| \| 437.9 \| ICD9 \|  \| Vascular \| \| 438.0 \| ICD9 \|  \| Vascular \| \| 438.10 \| ICD9 \|  \| Vascular \| \| 438.11 \| ICD9 \|  \| Vascular \| \| 438.12 \| ICD9 \|  \| Vascular \| \| 438.13 \| ICD9 \|  \| Vascular \| \| 438.19 \| ICD9 \|  \| Vascular \| \| 438.20 \| ICD9 \|  \| Vascular \| \| 438.21 \| ICD9 \|  \| Vascular \| \| 438.22 \| ICD9 \|  \| Vascular \| \| 438.30 \| ICD9 \|  \| Vascular \| \| 438.40 \| ICD9 \|  \| Vascular \| \| 438.53 \| ICD9 \|  \| Vascular \| \| 438.6 \| ICD9 \|  \| Vascular \| \| 438.7 \| ICD9 \|  \| Vascular \| \| 438.81 \| ICD9 \|  \| Vascular \| \| 438.82 \| ICD9 \|  \| Vascular \| \| 438.83 \| ICD9 \|  \| Vascular \| \| 438.84 \| ICD9 \|  \| Vascular \| \| 438.85 \| ICD9 \|  \| Vascular \| \| 438.89 \| ICD9 \|  \| Vascular \| \| 438.9 \| ICD9 \|  \| Vascular \| \| 440.0 \| ICD9 \|  \| Vascular \| \| 440.1 \| ICD9 \|  \| Vascular \| \| 440.20 \| ICD9 \|  \| Vascular \| \| 440.21 \| ICD9 \|  \| Vascular \| \| 440.22 \| ICD9 \|  \| Vascular \| \| 440.23 \| ICD9 \|  \| Vascular \| \| 440.24 \| ICD9 \|  \| Vascular \| \| 440.29 \| ICD9 \|  \| Vascular \| \| 440.30 \| ICD9 \|  \| Vascular \| \| 440.31 \| ICD9 \|  \| Vascular \| \| 440.4 \| ICD9 \|  \| Vascular \| \| 440.8 \| ICD9 \|  \| Vascular \| \| 440.9 \| ICD9 \|  \| Vascular \| \| 442.1 \| ICD9 \|  \| Vascular \| \| 443 \| ICD9 \|  \| Vascular \| \| 443.2 \| ICD9 \|  \| Vascular \| \| 443.21 \| ICD9 \|  \| Vascular \| \| 443.22 \| ICD9 \|  \| Vascular \| \| 443.24 \| ICD9 \|  \| Vascular \| \| 443.29 \| ICD9 \|  \| Vascular \| \| 443.89 \| ICD9 \|  \| Vascular \| \| 443.9 \| ICD9 \|  \| Vascular \| \| 707.10 \| ICD9 \|  \| Vascular \| \| 707.11 \| ICD9 \|  \| Vascular \| \| 707.12 \| ICD9 \|  \| Vascular \| \| 707.13 \| ICD9 \|  \| Vascular \| \| 707.19 \| ICD9 \|  \| Vascular \| \| G45.0 \| ICD10 \|  \| Vascular \| \| G45.1 \| ICD10 \|  \| Vascular \| \| G45.8 \| ICD10 \|  \| Vascular \| \| G45.9 \| ICD10 \|  \| Vascular \| \| I13.11 \| ICD10 \|  \| Vascular \| \| I13.2 \| ICD10 \|  \| Vascular \| \| I20.0 \| ICD10 \|  \| Vascular \| \| I20.1 \| ICD10 \|  \| Vascular \| \| I20.8 \| ICD10 \|  \| Vascular \| \| I20.9 \| ICD10 \|  \| Vascular \| \| I21.09 \| ICD10 \|  \| Vascular \| \| I21.11 \| ICD10 \|  \| Vascular \| \| I21.19 \| ICD10 \|  \| Vascular \| \| I21.29 \| ICD10 \|  \| Vascular \| \| I21.3 \| ICD10 \|  \| Vascular \| \| I21.4 \| ICD10 \|  \| Vascular \| \| I21.9 \| ICD10 \|  \| Vascular \| \| I21.A1 \| ICD10 \|  \| Vascular \| \| I24.0 \| ICD10 \|  \| Vascular \| \| I24.1 \| ICD10 \|  \| Vascular \| \| I24.8 \| ICD10 \|  \| Vascular \| \| I25.10 \| ICD10 \|  \| Vascular \| \| I25.2 \| ICD10 \|  \| Vascular \| \| I25.3 \| ICD10 \|  \| Vascular \| \| I25.41 \| ICD10 \|  \| Vascular \| \| I25.42 \| ICD10 \|  \| Vascular \| \| I25.5 \| ICD10 \|  \| Vascular \| \| I25.810 \| ICD10 \|  \| Vascular \| \| I25.811 \| ICD10 \|  \| Vascular \| \| I25.82 \| ICD10 \|  \| Vascular \| \| I25.83 \| ICD10 \|  \| Vascular \| \| I25.84 \| ICD10 \|  \| Vascular \| \| I25.89 \| ICD10 \|  \| Vascular \| \| I25.9 \| ICD10 \|  \| Vascular \| \| I50.1 \| ICD10 \|  \| Vascular \| \| I50.20 \| ICD10 \|  \| Vascular \| \| I50.21 \| ICD10 \|  \| Vascular \| \| I50.22 \| ICD10 \|  \| Vascular \| \| I50.23 \| ICD10 \|  \| Vascular \| \| I50.30 \| ICD10 \|  \| Vascular \| \| I50.31 \| ICD10 \|  \| Vascular \| \| I50.32 \| ICD10 \|  \| Vascular \| \| I50.33 \| ICD10 \|  \| Vascular \| \| I50.40 \| ICD10 \|  \| Vascular \| \| I50.41 \| ICD10 \|  \| Vascular \| \| I50.42 \| ICD10 \|  \| Vascular \| \| I50.43 \| ICD10 \|  \| Vascular \| \| I50.9 \| ICD10 \|  \| Vascular \| \| I60.9 \| ICD10 \|  \| Vascular \| \| I61.9 \| ICD10 \|  \| Vascular \| \| I62.00 \| ICD10 \|  \| Vascular \| \| I62.1 \| ICD10 \|  \| Vascular \| \| I62.9 \| ICD10 \|  \| Vascular \| \| I63.20 \| ICD10 \|  \| Vascular \| \| I63.219 \| ICD10 \|  \| Vascular \| \| I63.22 \| ICD10 \|  \| Vascular \| \| I63.239 \| ICD10 \|  \| Vascular \| \| I63.30 \| ICD10 \|  \| Vascular \| \| I63.40 \| ICD10 \|  \| Vascular \| \| I63.50 \| ICD10 \|  \| Vascular \| \| I63.59 \| ICD10 \|  \| Vascular \| \| I65.09 \| ICD10 \|  \| Vascular \| \| I65.1 \| ICD10 \|  \| Vascular \| \| I65.29 \| ICD10 \|  \| Vascular \| \| I65.8 \| ICD10 \|  \| Vascular \| \| I65.9 \| ICD10 \|  \| Vascular \| \| I66.09 \| ICD10 \|  \| Vascular \| \| I66.19 \| ICD10 \|  \| Vascular \| \| I66.29 \| ICD10 \|  \| Vascular \| \| I66.9 \| ICD10 \|  \| Vascular \| \| I67.1 \| ICD10 \|  \| Vascular \| \| I67.2 \| ICD10 \|  \| Vascular \| \| I67.4 \| ICD10 \|  \| Vascular \| \| I67.6 \| ICD10 \|  \| Vascular \| \| I67.81 \| ICD10 \|  \| Vascular \| \| I67.82 \| ICD10 \|  \| Vascular \| \| I67.848 \| ICD10 \|  \| Vascular \| \| I67.89 \| ICD10 \|  \| Vascular \| \| I67.9 \| ICD10 \|  \| Vascular \| \| I69.898 \| ICD10 \|  \| Vascular \| \| I69.90 \| ICD10 \|  \| Vascular \| \| I69.91 \| ICD10 \|  \| Vascular \| \| I69.910 \| ICD10 \|  \| Vascular \| \| I69.911 \| ICD10 \|  \| Vascular \| \| I69.915 \| ICD10 \|  \| Vascular \| \| I69.919 \| ICD10 \|  \| Vascular \| \| I69.920 \| ICD10 \|  \| Vascular \| \| I69.921 \| ICD10 \|  \| Vascular \| \| I69.922 \| ICD10 \|  \| Vascular \| \| I69.928 \| ICD10 \|  \| Vascular \| \| I69.931 \| ICD10 \|  \| Vascular \| \| I69.934 \| ICD10 \|  \| Vascular \| \| I69.939 \| ICD10 \|  \| Vascular \| \| I69.949 \| ICD10 \|  \| Vascular \| \| I69.951 \| ICD10 \|  \| Vascular \| \| I69.952 \| ICD10 \|  \| Vascular \| \| I69.953 \| ICD10 \|  \| Vascular \| \| I69.954 \| ICD10 \|  \| Vascular \| \| I69.959 \| ICD10 \|  \| Vascular \| \| I69.969 \| ICD10 \|  \| Vascular \| \| I69.990 \| ICD10 \|  \| Vascular \| \| I69.991 \| ICD10 \|  \| Vascular \| \| I69.992 \| ICD10 \|  \| Vascular \| \| I69.993 \| ICD10 \|  \| Vascular \| \| I69.998 \| ICD10 \|  \| Vascular \| \| I70.0 \| ICD10 \|  \| Vascular \| \| I70.1 \| ICD10 \|  \| Vascular \| \| I70.209 \| ICD10 \|  \| Vascular \| \| I70.219 \| ICD10 \|  \| Vascular \| \| I70.229 \| ICD10 \|  \| Vascular \| \| I70.25 \| ICD10 \|  \| Vascular \| \| I70.269 \| ICD10 \|  \| Vascular \| \| I70.299 \| ICD10 \|  \| Vascular \| \| I70.8 \| ICD10 \|  \| Vascular \| \| I70.90 \| ICD10 \|  \| Vascular \| \| I70.91 \| ICD10 \|  \| Vascular \| \| I70.92 \| ICD10 \|  \| Vascular \| \| I72.2 \| ICD10 \|  \| Vascular \| \| I73.9 \| ICD10 \|  \| Vascular \| \| I77.71 \| ICD10 \|  \| Vascular \| \| I77.72 \| ICD10 \|  \| Vascular \| \| I77.74 \| ICD10 \|  \| Vascular \| \| I77.79 \| ICD10 \|  \| Vascular \| \| L97.209 \| ICD10 \|  \| Vascular \| \| L97.309 \| ICD10 \|  \| Vascular \| \| L97.809 \| ICD10 \|  \| Vascular \| \| L97.909 \| ICD10 \|  \| Vascular \| | \| 35301 \| \| --- \| \| 37205 \| \| 33533 \| \| 33518 \| \| 021009W \| \| 02100A3 \| \| 02100A9 \| \| 02100AW \| \| 02100Z8 \| \| 02100Z9 \| \| 02100ZC \| \| 021109W \| \| 02110Z9 \| \| 021209W \| \| 021309W \| \| 03CH0ZZ \| \| 03CJ0ZZ \| \| 03CK0ZZ \| \| 03CL0ZZ \| \| 03CM0ZZ \| \| 03CN0ZZ \| \| 36.06 \| \| 36.07 \| \| 36.11 \| \| 36.12 \| \| 36.13 \| \| 36.14 \| \| 36.15 \| \| 38.12 \| |

Table 3. Sensitivity Analysis Results: Transition Intensity Matrix.

| **Transition** | **Original Population** | **All Micro** | **All Macro** |
| --- | --- | --- | --- |
| *Diabetes 🡪 Micro* | .1334 [.1284,.1386] | .1345 [.1298,.1401] | .1342 [.1292,.1394] |
| *Diabetes 🡪 Macro* | .0508 [.0479,.0540] | .0511 [.0481,.0543] | .0520 [.0490,.0552] |
| *Diabetes 🡪 Death* | .0001 [.0001,.0007 | .0001 [.0000,.0006] | .0001 [.0000,.0007] |
| *Micro 🡪 Both* | .0395 [.0355,.0439] | .0415 [.0374,.0460] | .0386 [.0345,.0430] |
| *Micro 🡪 Death* | .0014 [.0001,.0036] | .0014 [.0005,.0035] | .0021 [.0012,.0038] |
| *Macro 🡪 Both* | .0784 [.0679,.0905] | .0775 [.0671,.0895] | .0835 [.0727,.0960] |
| *Macro 🡪 Death* | .0134 [.0083,.0216] | .0026 [.0009,.0076] | .0032 [.0013,.0077] |
| *Both 🡪 Death* | .0023 [.0006,.0079] | .0131 [.0082,.0211] | .0109 [.0067,.0178] |

Transition intensity [95% CI].

Table 4. Sensitivity Analysis Results: Estimated 1-Year, 3-Year , and 5-Year State-to-State Transition Probabilities Among Diabetes Patients

| **Year 1 and Iteration** | **Maximum Likelihood Estimate** (95% CI) | | | | |  |  |
| --- | --- | --- | --- | --- | --- | --- | --- |
|  | Diabetes | Microvascular | Macrovascular | Both | Death |  |  |
| Original |  |  |  |  |  |  |  |
| Diabetes | 0.832 (0.827,0.836) | 0.119 (0.115,0.124) | 0.044 (0.042,0.047) | 0.004 (0.003,0.005) | 0.000 (0.000,0.001) |  |  |
| Microvascular | - | 0.960 (0.955,0.964) | - | 0.038 (0.035,0.042) | 0.002 (0.001,0.004) |  |  |
| Macrovascular | - | - | 0.923 (0.910,0.933) | 0.075 (0.065,0.086) | 0.003 (0.001,0.008) |  |  |
| Both | - | - | - | 0.987 (0.978,0.992) | 0.013 (0.008,0.022) |  |  |
| Death | - | - | - | - | 1.0000 |  |  |
| All Micro |  |  |  |  |  |  |  |
| Diabetes | 0.830 (0.825,0.835) | 0.120 (0.116,0.125) | 0.0448 (0.042,0.047) | 0.004 (0.004,0.005) | 0.000 (0.000,0.001) |  |  |
| Microvascular | - | 0.958 (0.953,0.962) | - | 0.040 (0.036,0.045) | 0.002 (0.001,0.004) |  |  |
| Macrovascular | - | - | 0.923 (0.911,0.932) | 0.074 (0.064,0.086) | 0.003 (0.001,0.008) |  |  |
| Both | - | - | - | 0.987 (0.979,0.992) | 0.013 (0.008,0.022) |  |  |
| Death | - | - | - | - | 1.0000 |  |  |
| All Macro |  |  |  |  |  |  |  |
| Diabetes | 0.830 (0.825,0.835) | 0.120 (0.116,0.124) | 0.045 (0.043,0.048) | 0.004 (0.004,0.005) | 0.000 (0.000,0.001) |  |  |
| Microvascular | - | 0.960 (0.956,0.964) | - | 0.038 (0.034,0.042) | 0.002 (0.001,0.004) |  |  |
| Macrovascular | - | - | 0.917 (0.905,0.926) | 0.079 (0.071,0.090) | 0.003 (0.002,0.008) |  |  |
| Both | - | - | - | 0.989 (0.983,0.993) | 0.011 (0.007,0.017) |  |  |
| Death | - | - | - | - | 1.0000 |  |  |
| **Year 3 and Iteration** | |  | | | | |  |
|  |  |  |  |  |  |  |  |
| Original | |  |  |  |  |  |  |
| Diabetes | | 0.575 (0.565,0.585) | 0.288 (0.278,0.296) | 0.103 (0.097,0.109) | 0.032 (0.030,0.035) | 0.002 (0.001,0.003) |  |
| Microvascular | | - | 0.885 (0.872,0.896) | - | 0.109 (0.099,0.121) | 0.006 (0.004,0.012) |  |
| Macrovascular | | - | - | 0.785 (0.754,0.810) | 0.205 (0.179,0.233) | 0.10 (0.006,0.024) |  |
| Both | | - | - | - | 0.961 (0.938,0.976) | 0.039 (0.024,0.062) |  |
| Death | | - | - | - | - | 1.0000 |  |
| All Micro | |  |  |  |  |  |  |
| Diabetes | | 0.572 (0.561,0.582) | 0.290 (0.280,0.298) | 0.103 (0.097,0.109) | 0.033 (0.031,0.036) | 0.002 (0.001,0.003) |  |
| Microvascular | | - | 0.879 (0.867,0.891) | - | 0.115 (0.103,0.125) | 0.006 (0.004,0.011) |  |
| Macrovascular | | - | - | 0.786 (0.757,0.811) | 0.203 (0.179,0.230) | 0.010 (0.006,0.023) |  |
| Both | | - | - | - | 0.961 (0.941,0.975) | 0.039 (0.025,0.059) |  |
| Death | | - | - | - | - | 1.0000 |  |
| All Macro | |  |  |  |  |  |  |
| Diabetes | | 0.572 (0.561,0.582) | 0.289 (0.279,0.298) | 0.104 (0.098,0.110) | 0.033 (0.031,0.036) | 0.002 (0.002,0.004) |  |
| Microvascular | | - | 0.885 (0.873,0.896) | - | 0.107 (0.097,0.119) | 0.008 (0.005,0.012) |  |
| Macrovascular | | - | - | 0.771 (0.743,0.796) | 0.217 (0.192,0.242) | 0.012 (0.007,0.024) |  |
| Both | | - | - | - | 0.968 (0.949,0.980) | 0.032 (0.020,0.051) |  |
| Death | | - | - | - | - | 1.0000 |  |
| **Year 5 and Iteration** | | |  | | | | |
|  |  |  |  |  |  |  |  |
| Original | | |  |  |  |  |  |
| Diabetes | | | 0.398 (0.387,0.410) | 0.388 (0.377,0.399) | 0.133 (0.124,0.140) | 0.077 (0.071,0.083) | 0.005 (0.004,0.008) |
| Microvascular | | | - | 0.815 (0.796,0.830) | - | 0.172 (0.157,0.190) | 0.012 (0.008,0.021) |
| Macrovascular | | | - | - | 0.668 (0.623,0.701) | 0.311 (0.277,0.348) | 0.021 (0.013,0.043) |
| Both | | | - | - | - | 0.935 (0.896,0.959) | 0.065 (0.041,0.104) |
| Death | | | - | - | - | - | 1.0000 |
| All Micro | | |  |  |  |  |  |
| Diabetes | | | 0.394 (0.383,0.406) | 0.388 (0.377,0.399) | 0.133 (0.124,0.140) | 0.079 (0.073,0.086) | 0.005 (0.004,0.008) |
| Microvascular | | | - | 0.807 (0.789,0.824) | - | 0.181 (0.164,0.197) | 0.012 (0.008,0.021) |
| Macrovascular | | | - | - | 0.670 (0.628,0.706) | 0.308 (0.272,0.347) | 0.021 (0.013,0.043) |
| Both | | | - | - | - | 0.936 (0.900,0.960) | 0.064 (0.039,0.100) |
| Death | | | - | - | - | - | 1.0000 |
| All Macro | | |  |  |  |  |  |
| Diabetes | | | 0.394 (0.382,0.405) | 0.389 (0.377,0.401) | 0.133 (0.124,0.141) | 0.079 (0.072,0.085) | 0.006 (0.005,0.008) |
| Microvascular | | | - | 0.816 (0.798,0.832) | - | 0.170 (0.153,0.186) | 0.014 (0.010,0.021) |
| Macrovascular | | | - | - | 0.648 (0.607,0.684) | 0.329 (0.293,0.366) | 0.023 (0.014,0.040) |
| Both | | | - | - | - | 0.947 (0.914,0.968) | 0.053 (0.032,0.086) |
| Death | | | - | - | - | - | 1.0000 |
